# Computerized Adaptive Testing Provides Reliable and Efficient Depression Measurement Using the CES-D Scale

**DOI:** 10.2196/jmir.7453

**Published:** 2017-09-20

**Authors:** Bao Sheng Loe, David Stillwell, Chris Gibbons

**Affiliations:** ^1^ School of Psychology University of Cambridge Cambridge United Kingdom; ^2^ The Psychometrics Centre Judge Business School University of Cambridge Cambridge United Kingdom; ^3^ Cambridge Centre for Health Services Research Cambridge School of Clinical Medicine University of Cambridge Cambridge United Kingdom

**Keywords:** depression, assessment, psychometrics, patient reported outcome measures, patient outcome assessment

## Abstract

**Background:**

The Center for Epidemiologic Studies Depression Scale (CES-D) is a measure of depressive symptomatology which is widely used internationally. Though previous attempts were made to shorten the CES-D scale, few have attempted to develop a Computerized Adaptive Test (CAT) version for the CES-D.

**Objective:**

The aim of this study was to provide evidence on the efficiency and accuracy of the CES-D when administered using CAT using an American sample group.

**Methods:**

We obtained a sample of 2060 responses to the CESD-D from US participants using the myPersonality application. The average age of participants was 26 years (range 19-77). We randomly split the sample into two groups to evaluate and validate the psychometric models. We used evaluation group data (n=1018) to assess dimensionality with both confirmatory factor and Mokken analysis. We conducted further psychometric assessments using item response theory (IRT), including assessments of item and scale fit to Samejima’s graded response model (GRM), local dependency and differential item functioning. We subsequently conducted two CAT simulations to evaluate the CES-D CAT using the validation group (n=1042).

**Results:**

Initial CFA results indicated a poor fit to the model and Mokken analysis revealed 3 items which did not conform to the same dimension as the rest of the items. We removed the 3 items and fit the remaining 17 items to GRM. We found no evidence of differential item functioning (DIF) between age and gender groups. Estimates of the level of CES-D trait score provided by the simulated CAT algorithm and the original CES-D trait score derived from original scale were correlated highly. The second CAT simulation conducted using real participant data demonstrated higher precision at the higher levels of depression spectrum.

**Conclusions:**

Depression assessments using the CES-D CAT can be more accurate and efficient than those made using the fixed-length assessment.

## Introduction

The Center for Epidemiologic Studies Depression Scale (CES-D) is a commonly used 20-item self-rating scale designed to measure depressive symptomatology in both clinical and non-clinical settings [1]. It is used in both epidemiological research and as a diagnostic screening tool [2,3].

Despite much debate on the cut-off score which yield better sensitivities and specificities [3,4], it is commonly accepted that persons who score 16 or above on the CES-D’s 0 to 60 scale are likely to be clinically depressed [5,6]. While some authors have suggested that the CES-D has a four-factor structure, it appears to provide meaningful measurement along a single dimension [7]. Hence, this level of internal consistency suggests that the CES-D should be used as an overall scale to measure a single latent construct—depressive symptoms [8].

Although the fixed-length version of the CES-D is widely used, recent developments in the availability of software to conduct advanced psychometric analyses and to develop computer adaptive assessments bring new opportunities for advanced Internet-based depressive symptom assessment. Computerized Adaptive Testing (CAT), refers to an algorithm-based assessment protocol which iteratively matches participants in a psychometric assessment with the most relevant item for them. Conducting assessments in this manner often reduces the number of items which need to be administered in an assessment, reducing the length of assessments by as much as 82%, compared to fixed-length measures of the same construct [9-11]. CAT typically relies on item parameter information derived from item-response theory. A large number of item-response theory models are suitable for developing item banks including the graded response model (GRM), the Rasch family of models as well as multidimensional models [12,13].

As well as demonstrable increases in efficiency, CATs can deal with other issues which prohibit accurate measurement using static questionnaires. For example, CATs are able to adjust for demographic differences in the interpretations of items commonly seen between different groups and known as differential item functioning (DIF) [14-16]. It is also possible to account for issues caused by items being *too* similar which can spuriously inflate assessment reliability [17].

An investigation conducted by Smit et al [10] demonstrated that the CES-D items make suitable candidates for CAT administration in a sample of Dutch adolescents aged between 12 and 17 [18]. The study shows that CAT administration could approach the reliability of the paper-based measures using fewer than half the items on the original. Other CATs have developed novel item banks to create CATs of depression, including the D-CAT [19,20] and PROMIS depression item banks [21]. These item banks show similar performance, arriving at reliable estimates of depressive symptomology using fewer than 10 items. Though both using legacy questionnaires to “feed” CATs and developing item banks specifically for that purpose have advantages and disadvantages. One advantages of using the CES-D for CAT is that it is not only well known and widely understood but it is also freely available in the public domain, allowing its use as a CAT assessment without incurring additional fees or reliance on restrictive proprietary software.

Thus, this paper aims to validate the CES-D assessment for use as a Web-based CAT using a sample taken from the US general population which will allow patients, clinicians, and other members of the public to evaluate depression symptomology efficiently and precisely online.

## Methods

### Participants

We recruited 2060 individuals who completed the CES-D scale via the myPersonality application [22]. MyPersonality is a Facebook application that allowed Facebook users to complete psychological tests and receive feedback on their scores. Users of the myPersonality application provided opt-in consent to allow us to record their assessment scores in exchange for the opportunity to receive feedback, which can be later shared online. The sample was divided into two groups using a randomly generated numeric string (random.org) for analysis. The first group is used for evaluation of the CES-D scale (n=1018). The second group is used for validating the CAT results based the calibration of the item parameters derived from the evaluation sample (n=1042). The samples were independent from one another. For group 1, there were 65.52% (665/1018) 6 females and 34.39% (348/1012) males. The mean age of the participants was 26 years (SD 12.12). For group 2, there were 65.93% (687/1042) females and 33.69% (351/1042) males. The mean age for participants was 25.86 (SD 10.44). Five participants from group 1 and 4 participants from group 2 did not reveal their gender. All individuals reported that they were from the United States.

### Measure

The CES-D is a self-report questionnaire which measures severity of depression from the perspective of the individual (see Multimedia Appendix 1). Subjects responded to the CES-D by indicating on a 4-point Likert-scale stating how often each depressive symptom occurred during the past week (0=rarely or none of the time, 1=some of the time, 2=much of the time, 3=most or all the time). The potential range of scores is from 0 to 60, with higher scores indicating higher levels of depressive symptomology.

The CES-D scale is a well validated and widely used instrument in many studies internationally [23-25]. Reliability and validity of the scale has been tested in both general and clinical populations [1]. Previous results show that the 20-item scale yields good internal consistency for the general population (Cronbach alpha=.85) and for a psychiatric population (Cronbach alpha=.90) [26]. Adequate test-retest reliability was found over 2 to 8-week period and 3 to 12-month period, respectively [26,27]. Convergent validity was supported by the significant correlations with other scales designed to assess depression symptoms [18,19,28,29]. The CES-D scale is available to use in the public domain and free to use without restriction.

### Data Analysis

The internal consistency of the CES-D scale was determined using the Cronbach alpha statistic [30], confirmatory factor analysis (CFA) was first performed to determine the structure of the model. The maximum-likelihood estimator was in the confirmatory analyses. Four fit indices were used in this study: chi-square statistics [31]; the Comparative fit index (CFI, [32]), the Tucker Lewis Index (TLI, Tucker and Lewis 1973), and the root-mean-square error of approximation (RMSEA, [33]). The chi-square statistics indicates whether the observed covariance matrix is similar to the predicted covariance matrix. However, the result is liable to bias in large sample sizes [34]. As such, other criteria such as absolute and comparative fix indices are used to evaluate the model. The CFI and TLI indices are the relative reduction in lack of fit of an observed model versus an independent model; with values of 0.90 or greater indicating an adequate fit [35]. For RMSEA [33], values less than 0.05 indicate good fit, and values greater than 0.10 as indication of poor fit of a model after accounting for degrees of freedom of the model.

Subsequently, Mokken analysis was used to provide further insight into the scale’s factor structure and the scalability of the items [36,37]. Following Mokken analysis, data were analyzed using GRM [38], which has been shown to be suitable for calibrating items for use as CAT assessments [39]. Item discrimination values ranging from 0.64 to 1.34 were considered to be moderately discriminative, and values 1.35 or greater are highly discriminative [40].

Following the protocol set out by the PROMIS investigators [41], we assessed the assumptions of GRM and made modifications, where necessary, to the scale to resolve breaches of model assumptions, which are detailed below.

Local independence of items was assessed using Yen’s Q3 method of correlated residuals. Item residual correlations above .20 were considered indicative of local dependence between items [42]. Different strategies exist for managing items with local dependency, which including removing the items from the scale completely or collapsing the items into a testlet.

The DIF analysis using the lordif package was conducted for age and gender groups to identify measurement biases between groups [43]. The lordif package utilizes ordinal logistic regression methods to calculate DIF [44]. DIF is observed when the probability of answering a specific item correctly is not the same for individuals with the same level of depressive symptoms but who belong to a different demographic group [15]. For example, male and female participants may both have equal levels of depressive symptoms, but if the certain items are interpreted differently between groups then observed mean scores may incorrectly show that one group has higher levels of depressive symptoms than the other because of an artefact of their gender that was not adequately controlled for within the test. Hence, DIF is used to identify items with unwanted bias and indicate that the same item sets and parameters might be needed for different diagnostic groups [16].

We conducted DIF analysis to assess item invariance with respect to age and gender. Two criteria were adopted in this study to detect meaningful DIF: changes in the beta [43] and the pseudo R-square [45]. Values ranging from 5% to 10% beta change and pseudo R-squared >.13 suggest that meaningful DIF exist for a particular item [43,45,46]. For our study, items with beta change of above >1% was flagged for DIF. We divided the sample into 2 groups based on the mean age (26 years) of the sample. Participants who were younger than mean age were placed in the first group (n=399) and those that were older than the mean age were placed in the second group (n=200). For gender groups, all the males were in the first group (n=348), whereas all the females were in second group (n=665). Participants who did not wish to reveal their gender (n=9) were excluded from the DIF analysis as there were too few to create an adequate additional group.

We evaluated the impact of DIF on the CES-D scores by recalibrating the items to the GRM model using the DIF-adjusted item parameters [47]. The person scores were recalculated based on these parameters. Finally, the strength of the association between the DIF-adjusted person score and original person score were evaluated using Pearson correlation. A high correlation would suggest that adjusting for DIF would make negligible differences in the person scores, and as such, could be ignored [48]. A low correlation between the DIF-adjusted person scores and original person scores suggest that the DIF makes a meaningful difference on the final scores and that group-specific parameters should be used when developing a CAT.

### Establishing Evaluation of CES-D CAT Simulation

Two simulations were conducted to evaluate the properties of the item pool and the CAT algorithm. The first simulation employed simulated responses from various levels of the latent trait derived from participants who completed the full CES-D scale to determine the average number of items that had to be administered.

The second simulations were respondents from the validation group and thus, the simulations were conducted using real data. The item parameter estimates used in the CES-D CAT were derived from the evaluation group. The validation sample used in this simulation did not overlap with the evaluation sample used to calibrate the item bank. Nevertheless, the individuals of this sample completed the same CES-D items that had been employed in the construction of the item bank. As such, responses to all items in the item bank were available. Both the respondents’ latent trait levels and responses to individual items were used to estimate the number of items needed to administer in a CAT. Correlations with the simulated CAT score and their scores derived from the full CES-D were obtained for both groups.

The maximum Fisher Information criterion was used for item selection [49,50]. The Bayesian modal estimation was used at the beginning of the CAT simulation to estimate ability [51]. This approach temporarily assumes that the ability of the test takers is normally distributed. Once a mixed response pattern is obtained, the normal distribution assumption is no longer requires and thus, a non-Bayesian maximum likelihood estimation is used [52]. Maximum likelihood estimation is subsequently used to estimate the final ability of the test taker [49]. The major advantage of using maximum likelihood estimation of ability is that it can account for all the information in the test taker’s responses in conjunction with the information available on each test item. The stopping rule for both simulations were set at SE≤0.32, which roughly corresponds to a reliability value≥0.90 [53].

### Software

Analyses were all conducted using the R Statistical Computing Environment [54]. Individual packages were loaded to conduct CFA (“lavaan,” [55]), Mokken (“mokken,” [56]) and item response theory (IRT) including CAT simulations (“mirt,” [57] and “catR” [Magis and Raîche, 2011]).

## Results

### Confirmatory Factor Analysis

Confirmatory factor analysis (CFA) was employed to investigate the unidimensionality of the CES-D scale. Table 1 lists the mean, standard deviation, and the factor loadings of the CES-D items, revealing no reason for concern about the multivariate distribution of the data. Therefore, the model was estimated using the maximum likelihood method. As shown, the factor loadings are above the recommended threshold of .3 (Kline, 2013).

Initial CFA results indicate a poor fit to the model (χ^2^_8.4_, *P*<.05; TLI=0.94; CFI=0.86; and RMSEA=0.09 (95% CI=0.08-0.09)).

### Unidimensionality

We used Mokken analysis to further explore the dimensional structure of the CES-D and identify the potential sources of multidimensionality identified with the CFA. The evaluation of item homogeneity is based on the Loevinger’s *H* coefficient [58]. Scalability is considered to be sufficient for both items and the scale where Loevinger’s *H* is equal to or greater than 0.30 [59]. We found that items 2, 11, and 15 displayed item coefficients of homogeneity<0.3. Hence, these items were eliminated from further analysis. This strategy was repeated and all the items were found to be above the recommended threshold, which conformed to a single dimension with Loevinger’s coefficient of homogeneity at a scale level of 0.43 (Table 2).

**Table 1:** Factor loadings and item descriptive statistics for the CES-D scale.

Item no.	Mean	SD	Factor loadings
q1	2.09	0.95	0.53
q2	1.94	1.04	0.40
q3	2.22	1.07	0.81
q4	2.34	1.06	0.58
q5	2.67	1.00	0.51
q6	2.42	1.04	0.85
q7	2.48	1.01	0.49
q8	2.43	0.99	0.56
q9	2.16	1.09	0.70
q10	2.09	1.03	0.54
q11	2.57	1.11	0.42
q12	2.31	0.94	0.72
q13	2.25	1.01	0.60
q14	2.76	1.07	0.69
q15	1.90	0.92	0.41
q16	2.36	0.98	0.71
q17	1.77	0.96	0.54
q18	2.59	0.99	0.80
q19	2.30	1.07	0.63
q20	2.51	1.01	0.58

**Table 2:** Loevinger’s coefficient of homogeneity at an item-level.

Item	Mean	Item H (H_i_)^a^	Standard Error	Dimensionality
1	2.09	0.38	0.02	1
3	2.22	0.55	0.01	1
4	2.34	0.41	0.02	1
5	2.67	0.38	0.02	1
6	2.42	0.57	0.01	1
7	2.48	0.35	0.02	1
8	2.43	0.39	0.02	1
9	2.16	0.49	0.02	1
10	2.10	0.39	0.02	1
12	2.31	0.50	0.02	1
13	2.25	0.42	0.02	1
14	2.76	0.48	0.02	1
16	1.90	0.49	0.02	1
17	2.36	0.40	0.02	1
18	1.77	0.55	0.01	1
19	2.60	0.43	0.02	1
20	2.30	0.41	0.02	1

^a^Scale H=0.45.

### Graded Response Model

Once we have established unidimensionality using Mokken analysis. We fitted the remaining 17 items to Samejima’s GRM (Table 3). The slope and threshold parameters in the GRM are used describe the relationship between each item and overall depressive symptom severity. The slope parameter reflects how well the items discriminate between respondents with or without depressive symptoms. The item discrimination values (alpha) ranged from a high of alpha=3.70 (item 5) to a relative low, but still strong, alpha=1.13 (item 7). The threshold parameter describes the endorsement of depressive symptoms, with larger values indicating greater levels of depressive symptoms. The thresholds for the lowest item category (b1) ranged from −3.31 (item 18) to 0.15 (item 17) on a z-score scale, indicating low to average levels of depressive symptoms, relative to the rest of our sample, for the individuals who endorsed the lowest CES-D category. The thresholds for the highest CES-D category (b3) ranged from 3.50 (item 3) to 1.25 (item 14), indicating moderate to high levels of depressive symptoms. All the standard errors of the *b* estimates were considered marginal, indicating that the items were normally distributed. An item fit analysis was conducted to identify any misfits. However, the results indicated that the remaining items fitted the model. Examination of the factor loadings revealed that all items loaded significantly (>.50) on the single factor. Therefore, this model described the data adequately.

### Local independence

Local dependency was apparently between items 18,12, and 16 as well as items 1, 19, and 15. Items 8, 12, and 16 were grouped as testlet 1, and items 19, and 15 were grouped as testlet 2. We observed the item residual correlation and found that item 4 was still correlated (>0.2) with the first testlet. Hence, we grouped item 4 together with the first testlet and repeated the analysis, resulting in no correlated residuals greater than 0.2. Within the IRT framework, the fit indices based on the limited information M_2_ statistic was used to assess the model fit [60]. The result shows that the RMSEA was at 0.065 (95% CI 0.06-0.07), and comparative indices (TLI=0.96, CFI=0.97) were above the recommended threshold [35].

**Figure 1:**
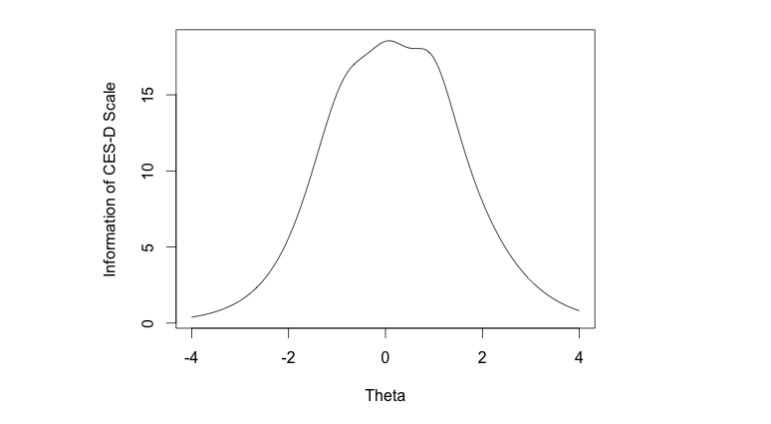
The test information of the CES-D scale.

Figure 1 displays the test information curve for the IRT GRM. The test characteristics curve is simply the additive of the scores associated with increasing levels of depressive symptoms. The test information is at its highest (18.71) when the theta level is slightly above 0, while the lowest around of information can be found at both tails of the *x*-axis. Hence, the CES-D scale is most precise in estimating the underlying trait when the theta level is approximately zero (average).

**Table 3:** Parameter estimates and factor loadings for the 17 items of the CES-D Scale.

Item	a	b1	b2	b3	Factor 1
Item 1	1.22 (0.09)	−0.97 (0.09)	0.93 (0.09)	2.85 (0.15)	0.58
Item 3	3.15 (0.18)	−1.56 (0.15)	1.14 (0.15)	3.50 (0.21)	0.88
Item 4	1.45 (0.09)	−1.31 (0.10)	0.20 (0.09)	2.16 (0.12)	0.65
Item 5	1.14 (0.08)	−2.13 (0.15)	−0.38 (0.08)	1.44 (0.10)	0.56
Item 6	3.70 (0.22)	−2.81 (0.21)	0.37 (0.16)	3.70 (0.24)	0.91
Item 7	1.13 (0.07)	−1.71 (0.10)	0.04 (0.08)	1.81 (0.10)	0.55
Item 8	1.37 (0.09)	−1.84 (0.11)	0.19 (0.09)	2.11 (0.12)	0.63
Item 9	2.03 (0.12)	−0.81 (0.11)	0.86 (0.11)	2.66 (0.15)	0.77
Item 10	1.29 (0.09)	−0.66 (0.09)	0.79 (0.09)	2.56 (0.13)	0.60
Item 12	2.14 (0.13)	−2.09 (0.15)	0.65 (0.12)	3.33 (0.18)	0.78
Item 13	1.49 (0.09)	−1.27 (0.10)	0.58 (0.09)	2.49 (0.13)	0.66
item 14	1.98 (0.11)	−2.59 (0.14)	−0.70 (0.11)	1.25 (0.11)	0.76
Item 16	2.12 (0.13)	−2.06 (0.14)	0.44 (0.12)	2.98 (0.16)	0.78
Item 17	1.38 (0.10)	0.15 (0.09)	1.57 (0.11)	3.24 (0.18)	0.63
Item 18	2.89 (0.16)	−3.31 (0.20)	−0.34 (0.14)	2.82 (0.18)	0.86
Item 19	1.50 (0.10)	−1.21 (0.10)	0.41 (0.09)	2.15 (0.12)	0.66
Item 20	1.37 (0.09)	−1.99 (0.12)	0.09 (0.09)	1.83 (0.11)	0.63

**Table 4:** CES-D CAT Simulation of respondents.

Measure	D1^a^	D2	D3	D4	D5	D6	D7	D8	D9	D10
Mean theta	−1.70	−1.04	−0.69	−0.37	−0.09	0.16	0.40	0.68	1.01	1.64
RMSE^b^	0.29	0.35	0.31	0.33	0.32	0.32	0.30	0.32	0.34	0.34
Mean bias	0.01	0.00	0.02	0.00	0.02	0.02	−0.03	−0.05	−0.07	0.01
Mean test length	13.98	12.34	11.04	9.50	8.30	7.70	7.60	8.18	9.77	13.09
Mean standard error	0.34	0.34	0.33	0.34	0.33	0.33	0.33	0.34	0.34	0.34
Number of simulees	105	104	104	104	104	104	104	104	104	105

^a^D: decile.

^b^RMSE: root mean square error

### DIF Analysis

DIF was not found between age groups. However, results indicated that item 14 (“I felt lonely”) showed moderate DIF for gender groups, with a beta change of more than 1% and a pseudo R-square of 0.08. When the DIF-adjusted person scores were calculated, the Pearson correlation between the original person scores and the DIF-adjusted person scores were 0.99. A *t* test analysis showed a non-significant mean difference with scores for DIF-adjusted person scores (mean=0.01, SD=0.88), and original person scores (mean=0.00, SD=0.97); *t*_2016.4_=−0.15, *P*=.88.

On the basis of these results, the conclusion arrived at was that statistically significant DIF was identified for item 14 using the two criteria of beta change and pseudo R-squared. However, the strength of association between the original person scores and the DIF-adjusted person scores were greater than 0.99. Therefore, the final decision was that any DIF found between the groups could be disregarded.

### Computer Adaptive Testing Simulation

#### Simulation I

Table 4 presents the results of the first simulation. In this analysis, the data were sorted into 10 equal parts, with each part representing one tenth of the sample group. There are appropriate 104 or 105 participants in each decile (D) rank (n=1042). The estimated average test length was 10.16 with SD of 2.34. The mean RMSE was .32 and the mean bias was −.0083. The lowest number of items administered to the simulees was in D5, with an average of 8.3 test items. The lowest and highest decile rank requires substantially more items (D1=13.98 items; D10=13.09 items) in order to reach the same target precision of SE≤0.32.

#### Simulation II

The second simulation study was conducted using the responses from a sample of real respondents (validation group) who completed the full CES-D scale. The stopping rule was set at SE≤0.32. The result of the second simulation can be found in Table 5. On average, 11.72 items with SD of 2.68 were required to estimate the latent trait at this level of precision. The mean RMSE was 1.14 and the mean bias was 0.18. Unlike the first simulation, only respondents in the lowest decile ranking required the administration of substantially more items to reach the specified level of precision (D1=14.76 items). Interestingly, there is a downward trend in the length of items from D9 (14.61 items) to D10 (8.41 items), indicating higher precision at higher levels of depressive symptoms with the use of lesser items.

**Figure 2:**
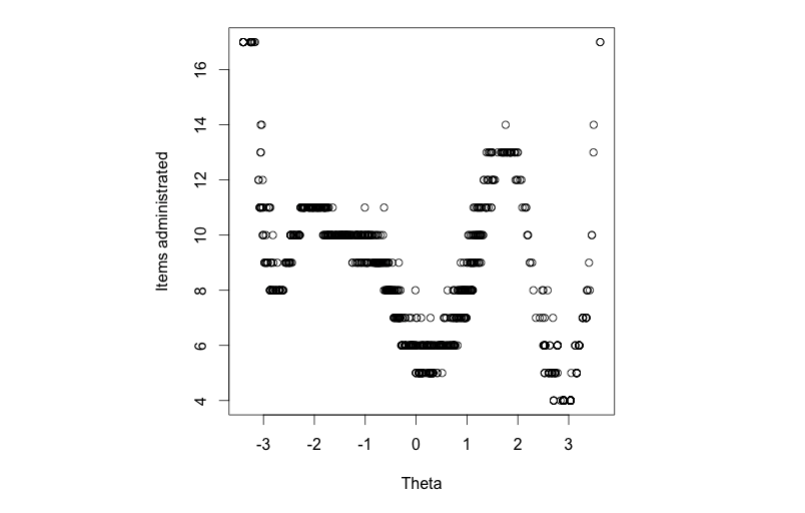
Relationship between number of items administered and level of depression (theta).

Further inspection of the item administration pattern (Figure 2), suggests a drop in the number of items required to estimate the latent trait accurately around. This could be due of the CAT algorithm selecting items with the highest information at every step, resulting in a quicker estimate of the latent trait. Figure 2 shows the number of items administered by the CES-D CAT as a function of the standardized score of the depressive symptoms construct.

**Figure 3:**
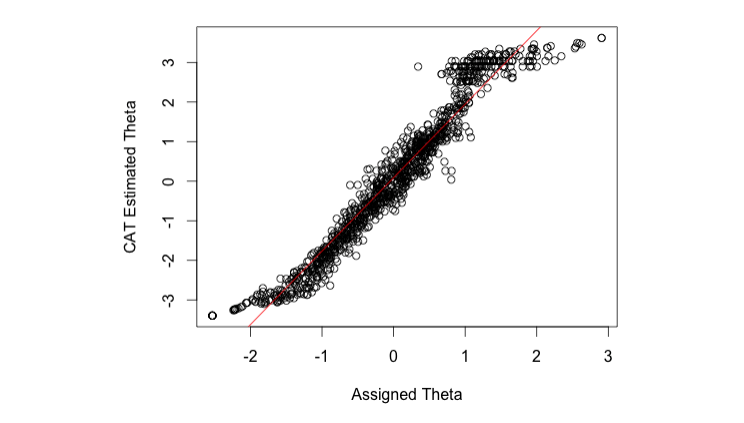
Comparison of CES-D CAT scores with an IRT score computed from all items in the item bank.

Estimates of the level of CES-D trait score provided by the simulated CAT algorithm and the original CES-D trait score derived from original scale correlated highly (*r*=0.98). This indicates that a precise estimation of the latent trait is possible with substantial item savings using CAT approaches (Figure 3).

Figure 3 shows exceptionally high correlation between the score from the CAT and the score given to the same participants when every item was completed.

**Table 5:** CES-D CAT Simulation of CAT algorithm.

Measure	D1	D2	D3	D4	D5	D6	D7	D8	D9	D10
Mean Theta	−1.70	−1.04	−0.69	−0.37	−0.09	0.16	0.40	0.68	1.01	1.64
RMSE	1.46	1.15	0.86	0.42	0.36	0.50	0.61	0.92	1.65	2.08
Mean bias	−1.45	−1.13	−0.82	−0.29	0.11	0.34	0.53	0.85	1.58	2.05
Mean test length	14.76	14.04	13.44	12.81	9.67	8.17	8.70	12.55	14.61	8.41
Mean standard error	0.42	0.34	0.33	0.33	0.33	0.33	0.34	0.34	0.33	0.37
Number of simulees	105	104	104	104	104	104	104	104	104	105

## Discussion

### Principal Findings

The psychometric properties of the CES-D measure were evaluated using a US sample. This sample was chosen to avoid issues of DIF across culture, and with the aim of providing an item bank which could be suitable for use within a clinical and research setting in the United States. The CES-D scale displayed excellent internal consistency based on the Cronbach alpha. The factor structure of the CES-D was subsequently evaluated using confirmatory factor and Mokken analysis. However, the results from the CFA indicated that the model provided an inadequate fit to the data. Mokken analysis identified three items as sources of multidimensionality in the CES-D. Items 2, 11, and 15 were considered to have poor fit and were subsequently removed from the analysis. Item 2 referred to “I did not feel like eating; my appetite was poor,” item 11 referred to “my sleep was restless,” and item 15 referred to “People were unfriendly.” The result showed that the final 17 items were found to be suitable for measuring a unidimensional trait and thus, the item parameters achieved from the remaining items allowed us to develop a computerized adaptive CES-D assessment.

The CES-D scale was calibrated using the IRT approaches. Most IRT based models require that items measure a single underlying dimension and this condition was met based on the result of the Mokken analysis. Furthermore, IRT based frameworks made computer-adaptive CES-D possible with the estimated item parameters derived from IRT models. Simulated computer-adaptive administration of the item bank demonstrates the ability to estimate precise latent trait levels with similar or higher levels of internal reliability similar to the original scale but using fewer items. These results are commensurate with other research exploring the performance of the CES-D as a CAT in other contexts including adolescents and people with multimorbidity [10,61] and for adaptive testing of depressive symptoms using the PROMIS system [62].

Unlike a test developed using classical test theory, in which the number of items is fixed and precision naturally varies between participants who have differing levels of latent ability, CAT fixes the precision while allowing the number of items to vary. CAT can only be conducted using computer administration and the items are previously calibrated with a suitable item response model. The steps to conducting a CAT are (1) administer an item, (2) compute the latent score and its standard error, (3) identify the next most informative item based on the current latent score estimate and IRT parameters, and (4) repeat steps 1-3 until the predefined stopping rule has been met.

In our simulation studies, we found a very high correlation between the CAT scores obtained when all 17 items were administered and when the stopping rule was introduced (leading to a mean test length of 10 items). Moreover, at the extreme (higher) end of the latent trait continuum, it only requires 8 items to identify individuals with depressive symptoms. This encourages quicker assessment of depressive symptoms, which can help clinicians to identify potential groups of persons who may benefit from immediate medical intervention. In spite of substantial improvements in efficiency by employing the CAT procedure, little information is lost and scores are still estimated accurately. By comparison, the time taken to complete the CES-D CAT will be shorter than the original 20-item scale. This time saving may seem small as far as a single scale is concerned, but psychometric assessment usually involves multiple questionnaires and, from this perspective, substantial time saving is evident.

### Limitations and Future Research

A limitation to the current research is the small number of items used to measure CES-D. With CAT, the precision of latent trait estimates increases with the number of items in the item bank. A smaller item bank gives fewer options for item selection and may results in reduced item variation between assessments. However, to apply stricter stopping-rule criteria means that the number of items necessary to complete the CES-D CAT will be about the same as completing all the original scale, thus, no extra benefit remains with the use of CES-D CAT. Therefore, while this study reports the stopping rule at less than or equal to 0.32, which is equivalent to a reliability of more than or equal to 0.90, the precision can still be heightened by increasing the test information. This can be achieved by adding more high-quality items to the item bank. Hence, future studies could evaluate the CAT system where new items are included as part of the test to increase the item bank and ensure that the performance of the CAT system is not compromised. The performance of the CAT algorithms can also be evaluated under “live” testing conditions rather than simulation of existing data to ensure that participants’ test performance under conventional ‘fixed length’ and adaptive conditions do not differ significantly.

Compared with population-based samples used in the development of item banks elsewhere [21], our sample was younger and had a greater proportion of women. Given the nature of the recruitment into the study via a voluntary online app it is not surprising that this sample is more reflective of a “digitally native” population of younger people. One important caveat of this research is therefore that our findings should not be extrapolated to a general population but rather support the growing body of literature demonstrating the suitability of the CES-D for adaptive testing in different group as a means of making measurement more precise and efficient while retaining an item bank that is familiar to clinicians.

In this study, we assess the content validity of the CAT-administered CESD by comparing depressive symptom estimates from the full-length assessment with an adaptively administered version. Further research is required to establish to predictive validity of this tool for the correct classification of clinical depression to support its use in clinical contexts.

There are some discussions about the factor structure of the CES-D and whether a single factor is appropriate for assessing depressive symptomatology. Several researchers have suggested that the CES-D scale is a measure of the underlying 4-factor structure [1,63,64]. However, the construction of a 4-factor scale may be too challenging as psychometric test designed for health assessment aims to be as short as possible. Nevertheless, in the event that a 4-factor scale is developed for the CES-D, then a 4-factor CES-D CAT under the conditions of content balancing may be introduced. In other words, a proportionate sampling of items is taken from each of the factor domains, while ensuring unidimensionality is achieved [52]. Researchers can thus consider new research avenues in which one could understand in finer gradient of the depressive symptoms.

### Conclusions

Our findings presented in this study shows that the CES-D CAT is a precise and efficient tool for screening depressive symptoms. Furthermore, the measurements provided by CAT are more likely to result in more meaningful research conclusions than classical approaches. More informed decisions could also be made based on measurement data at an individual level rather than at a scale level.

While increased complexity with regards to the test development is inevitable, the CES-D CAT has immediate advantages such as increased accuracy, exact interpretable, and shorter time spent over conventional testing approaches. Open source software such as the *Concerto* testing platform [65] makes it more accessible than ever before for researchers to develop and implement their own CAT system. Furthermore, the CESD-CAT outperforms the paper-based versions of the CES-D in terms of reliability, length, and flexibility in which they may be administered in a clinical setting. CATs are more dynamic as they adjust accordingly to the ability level of the test taker, indicating both efficiency and effectiveness. Thus, the CES-D CAT is suitable to be administered as a primary tool for understanding and screening individuals in the US with depressive symptomatology.

## Appendix

Multimedia Appendix 1 CES-D questions.

## Abbreviations

CAT: Computerized Adaptive Test
CES-D: Center for Epidemiologic Studies Depression Scale
CFA: Confirmatory factor analysis
CFI: Comparative fit index
DIF: Differential item functioning
GRM: graded response model
IRT: item response theory
RMSEA: root-mean-square error of approximation
TLI: Tucker Lewis Index
