# Supplementary material for: Computerized Adaptive Testing Provides Reliable and Efficient Depression Measurement Using the CES-D Scale
Source: J Med Internet Res. 2017 Sep 20;19(9):e302. doi: 10.2196/jmir.7453 (PMC5628285; doi:10.2196/jmir.7453)
Supplement: Multimedia Appendix 1 [file jmir_v19i9e302_app1.pdf]

1. I was bothered by things that usually don’t bother me
2. I did not feel like eating; my appetite was poor
3. I felt that I could not shake off the blues even with help from my family and friends
4. I felt that I was just as good as other people
5. I had trouble keeping my mind on what I was doing
6. I felt depressed
7. I felt that everything I did was an effort
8. I felt hopeful about the future
9. I thought my life had been a failure
10. I felt fearful
11. My sleep was restless
12. I was happy
13. I talked less than usual
14. I felt lonely
15. People were unfriendly
16. I enjoyed life
17. I had crying spells
18. I felt sad
19. I felt that people disliked me
20. I could not get “going”
